# School performance in youth after a concussion

**DOI:** 10.3389/fspor.2022.1008551

**Published:** 2022-12-22

**Authors:** Carol A. DeMatteo, Josephine Jakubowski, Sarah Randall, Kathy Stazyk, Chia-Yu Lin, Rebecca Yakubov

**Affiliations:** ^1^ School of Rehabilitation Sciences, McMaster University, Hamilton, ON, Canada; ^2^ CanChild Centre for Childhood Disability Research, McMaster University, Hamilton, ON, Canada

**Keywords:** return to school, pediatric concussion, return to activity, brain injury, school performance, children and youth

## Abstract

**Objective:**

This study aimed to identify school problems and levels of cognitive activity in youths aged 5–18 years with a concussion during the recovery stages of return to school (RTS).

**Study Design:**

In a prospective cohort, participants completed in-person assessments at three time points: First Visit Post-injury, Symptom Resolution Visit, and Follow-Up Visit. These time points varied based on the participants’ recovery progress. The post-concussion symptom scale (PCSS) and a cognitive activity scale were completed every 2 days until symptom resolution was achieved. Participants and their parents completed a school questionnaire detailing how their concussion had impacted their school learning/performance and their level of concern about their injury as well as the Immediate Post-Concussion Assessment and Cognitive Testing (ImPACT).

**Results:**

Sixty-three percent (*N* = 44/70) of participants returned to school by the First Visit Post-injury (average 7.7 days following injury), and of these, 50% (*N* = 22) were experiencing school problems. Sixty-five participants (out of 70) returned to school at the Follow-Up Visit, and of these, 18% reported school problems. There was a significant difference in the school problems reported by parents and youth. At the First Visit Post-injury, the youth reported more problems (*p *= 0.02), and the In-Person Symptom Resolution Visit with parents reported more problems (*p *= 0.01). The cognitive activity score increased, while the PCSS score decreased from RTS Stage 1 to Stage 5.

**Conclusions:**

This study identified that 50% of youth experienced school problems at the First Visit Post-injury, whereas only 18% reported school problems at the Follow-Up Visit. There is a significant difference in the perception of school problems reported by youth and their parents at different stages of recovery. The amount and complexity of cognitive activity increased with decreasing symptoms and increasing RTS stage. Findings can guide youth with a concussion and their parents in supporting a cautious return to school with accommodations. Healthcare providers and researchers can use this knowledge to better support youth in their return to school and understand the importance of gathering information from youth and their parents to gain the best insight into recovery.

## Introduction

In Ontario, the annual incidence of concussion is 1% of the population, with children having the highest incidence (3,600 per 100,000 individuals) (1, 2). A concussion has detrimental impacts on a child's physical, psychological, and academic performance (3–5), affecting school attendance (6), participation in sports or social activities (7), and quality of life (8–10). Although many children become symptom-free within 2 weeks, approximately one-third of children experience prolonged symptoms past 1 month, referred to as persistent post-concussion symptoms (PCS) (11, 12). For clarity, we will be referring individuals aged 5–18 years as youth in the context of our study.

To help guide youth in their recovery from concussion, our research team developed evidence-based return to activity (RTA) and return to school (RTS) protocols (13, 14). These protocols [now updated (15)] are based on the Sports Concussion Consensus statements (16, 17), highlighting best practices in managing concussion recovery. The importance of returning to school cautiously is emphasized in current post-concussion recovery protocols (18). Our group recently observed that youth (aged 5–18 years) return to school faster than they return to activity (35 and 38 days, respectively) and that 21% of youth were symptomatic at the full return to school (19). Similarly, Baker et al. (20) observed that one-third of concussed youth (defined in their paper as 13–19 years old) reported experiencing concussion-related symptoms and/or difficulties after returning to school (20).

Recently, Russell et al. (6) observed that following a sports-related concussion, children missed a median of 4 days of school and experienced a 1.0% decrease in their overall grade-point average (6). Upon a child's return to school, stressors such as missed activities (21) and a new curriculum can leave a child feeling overwhelmed (6), thus decreasing their academic performance (20). Furthermore, school problems and lowered grades may amplify a child's feelings of social isolation and lower their self-esteem (22). As such, it is important to determine a youth's perceived school problems following a concussion. Furthermore, it is important to assess whether youth and parents differ in their perception of a youth's school problems and concerns following a concussion.

At the outset of this study, we hypothesized that youth would experience school problems following a concussion and that cognitive activity would increase over time as symptoms resolve. The specific aims of this research were to (1) examine youths’ perception of school problems and characterize school problems experienced by youth and (2) determine the concerns related to school and academic performance of youth with a concussion and their parents. The secondary objective was to examine the cognitive activity reported by participants (23) and changing patterns of symptom scale (post-concussion symptom scale, PCSS) (24) scores as youth progress through the RTS guidelines.

## Materials and methods

### Study design

Participants from the Back2Play Study were recruited between November 2014 and December 2016 from the McMaster Children's Hospital Emergency Department, community referrals from primary physicians, and sports medicine clinics in Hamilton, Ontario, Canada. Participants were eligible if they were 5–18 years old, had a confirmed diagnosis of a concussion from a physician, and were symptomatic at the time of recruitment. The median time from injury to study enrolment was 7.8 days (mean: 34.8 days, minimum: 2.9 h, and maximum: 320.9 days). Those with other injuries were allowed to participate, including those with nonaccidental injuries. Youth were considered ineligible if they were unable to speak English, had a significant brain injury requiring resuscitation, were admitted to the pediatric critical care unit, and/or required surgical intervention. Informed verbal and written assent and consent were obtained from participants and their parents. The study was approved by the Hamilton Integrated Research Ethics Board (#14-376).

This prospective longitudinal cohort study had three measurement time points. Participants attended two or three in-person assessments. Some participants only had First Visit and a Follow-Up Visit because their symptoms resolved in under 28 days (*N* = 51). For participants whose symptoms lasted longer than 28 days, they had three visits, with the third visit being 3 months post symptom resolution.

Following recruitment, participants wore an ActiGraph (ActiGraph LLC, Pensacola, FL, United States) to record the amount of movement and sleep (reported elsewhere) (25) and completed surveys every 48 h using REDCap (26) (Research Electronic Data Capture, a browser-based data management application). The surveys included the (1) PCSS (24), a common concussion evaluation consisting of a 22-symptom checklist scored on a 0–6 severity scale (where 0 denotes “not experiencing symptom” and 6 denotes “severely experiencing symptom”), (2) a self-report of the stage of RTA/RTS guidelines, and (3) a cognitive activity scale, adapted from Brown et al. (23) (Table 1). The PCSS score and cognitive scale score were assessed at the RTS stage entry and exit. Stage entry was defined as the first of two consecutive reports of a stage, whereas stage exit was reported as the last report of a stage. The time spent in each stage varied per participant.

**Table 1:** Cognitive scale score.

Cognitive scale score	
1	None—no reading, homework, text messaging, or screen time
2	Minimal—no reading or homework. Five text messages and 20-min per day screen time per day
3	Moderate—reading 10 pages, 20 text messages, 1-h combined homework and screen time per day
4	High—less reading, homework, and screen time than normal
5	Full—no restrictions to cognitive activity

Cognitive scale was adapted from Brown et al. (23).

At each in-person visit, participants completed the Immediate Post-Concussion Assessment and Cognitive Testing (ImPACT) reported here and quality of life and balance tests that are reported elsewhere (27–29). This manuscript aimed to depict school performance, concerns, and description of the amount and type of cognitive activity in youth during recovery from a concussion.

### Pediatric concussion recovery guidelines

RTS stages were participant-reported. Standard guidance and an explanation of the guidelines were provided at the outset of the study. Participants were given printed copies of the guidelines for reference. Participants progressed to the next stage after 48 h in any given stage without an increase in symptoms. Participant stage progression was self-determined**.** During Stage 1 of RTS, youth and their families were informed that “rest” does not equal social isolation, but rather youth were encouraged to participate in home/leisure activities as tolerated without worsening symptoms (i.e., make their bed, walk around the house) (17). Youth were also advised that they should begin getting ready to get back to school gradually, and if symptoms persist, they must progress to returning to school with accommodation. At Stage 2 of the RTS guidelines, youth were encouraged to begin simple cognitive activity at home for a maximum of 30 min (15 min of screen time twice daily, begin reading) without aggravating symptoms (17). At Stage 3, youth were encouraged to build up their back-to-school routines by increasing cognitive activity in a school environment with accommodations (17). These accommodations could be within their timetable, curriculum choices, environmental modifications, and adjustments in school activity participation as dictated by the individual child's needs and symptom profile. By RTS Stage 4, it was recommended that youth return back to full days of school, attending less than 5 days a week if needed (17). Youth were also encouraged to complete as much homework as possible and complete a maximum of one test per week. Finally, at Stage 5, the final stage of RTS, youth gradually returned to normal school routines (17). Although the focus of this manuscript is on cognitive activity and problems, it is important to note that participants were also following the RTA stages for physical activity simultaneously.

### School information and performance questionnaires (child)

**Figure 1:**
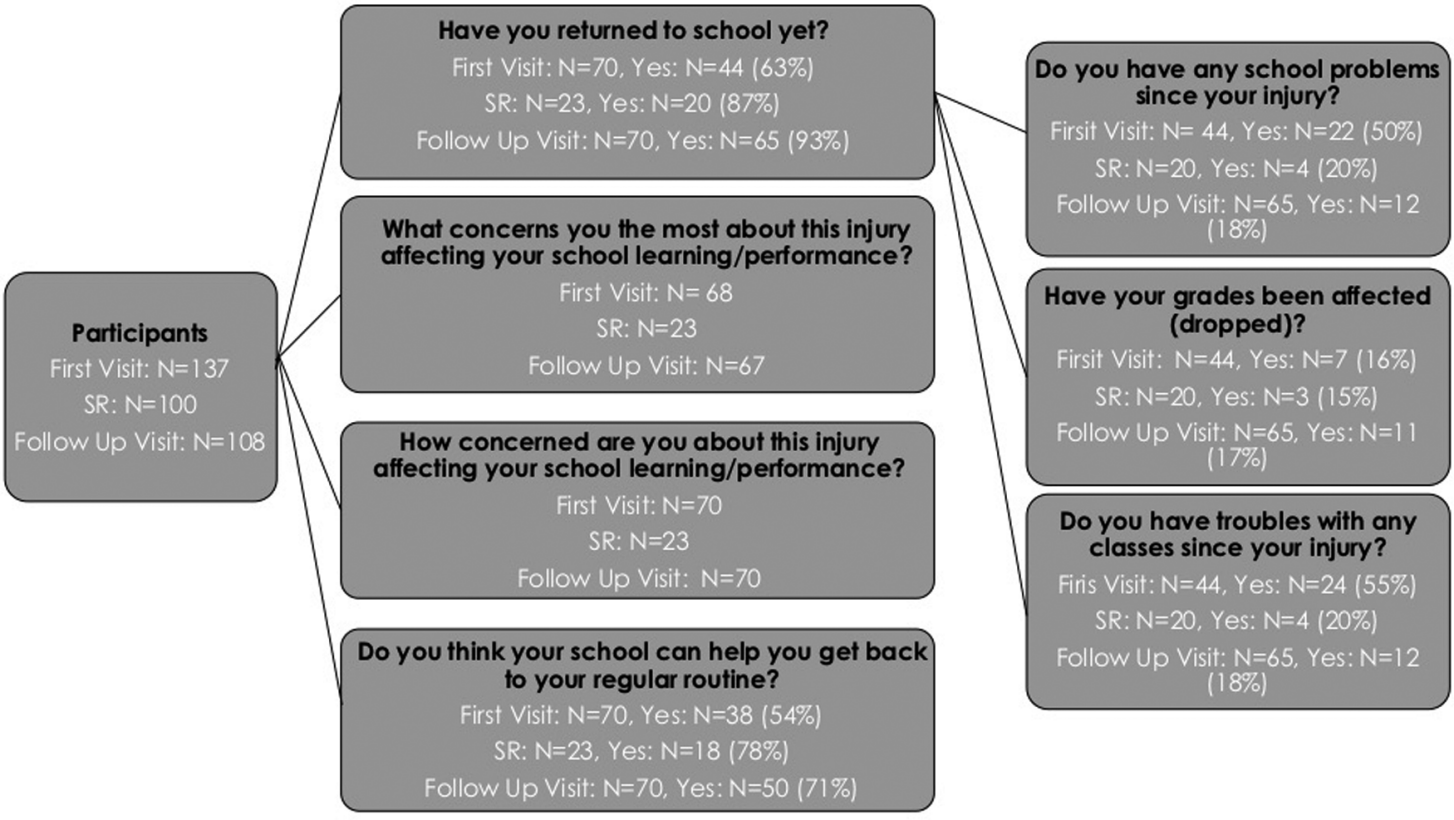
REDCap Question stems. Participants attended 3 in person assessments: First visit post injury, In Person Symptom resolution (if applicable), and Follow Up visit which occurred 3 months post symptom-resolution or 6 months post enrolment, whichever came first. Only participants recruited aged 9.5-18 years of age, recruited after July 2015, completed a school performance and information questionnaires. One hundred participants achieved symptom resolution within the study period, only 44 attended in person visits and were delivered the school information questionnaire.

Participants aged 9.5–18 years, recruited after July 2015, completed school performance and information questionnaires, reporting on symptoms and experiences that occurred within the “past week” (Figure 1). The questionnaire focused on school problems, defined as new problems or existing problems that have gotten worse since injury, as well as school concerns, which examined areas of concern, as well as the degree of concern. The participants were first asked the following questions: *Have you returned to school yet?* Participants responded “not applicable” if they were on holiday/summer vacation. *Only* if participants responded “yes” they were asked “*Do you have any school problems since your injury (either new or existing problems that have gotten worse since the injury)?*”. If participants responded yes to this question, branching logic provided the question, “*What kinds of school problems are you having SINCE YOUR INJURY? Check ALL that apply, either NEW problems or existing problems that have gotten WORSE since the injury*”. Questions regarding class troubles/difficulties, vulnerable times of day, and grades affected were also posed to participants who reported school problems. Participants were also asked, “*Have your grades been affected?*” and “*Do you have troubles with any classes/subjects since your injury?*” (Figure 1). For these questions (classes posing difficulties, vulnerable times of day, grades affected, how can your school help), participants could select *multiple* answers. Participants who had not yet returned to school or were at school but had not experienced new or worsening school problems were not asked these subsequent branching series of questions.

### School information and performance questionnaires (parent)

At each in-person visit, parents completed a report of their child's school problems. These questions included: “*How many school days has your child missed since his/her injury (since the last survey)?*”, “*Does your child have troubles with any classes/subjects since the injury?*”, and “*How concerned are you (not concerned, mildly, moderately, very) about this injury affecting your child's school learning and performance?*”. Note that the survey question related to missed school days did not explicitly ask parents to cite days missed due to concussion alone.

### Statistical analysis

Descriptive statistics were performed for all data. Data were assessed for normality; if data were not normally distributed, the median was reported. To assess our first aim, examining youths’ perception of school problems, youth-reported school problems are reported as percentages and proportions of participants at the First, Symptom Resolution, and Follow-Up Visit. Our second aim, youth- and parent-reported school concerns, is also reported as percentages and proportions of participants expressing school concerns at the First, Symptom Resolution, and Follow-Up Visit. To examine the proportion of youth and parents who reported school concerns and problems, chi-square tests were performed. Median time from injury to the First Visit Post-injury and the number of missed school days were reported as mean ± standard deviation. As for our secondary aims, to test the difference between the cognitive scale scores at the entry and exit of each stage of the RTS guidelines, a Wilcoxon signed-rank test was performed. All data were analyzed using SAS version 9.4 and SPSS Statistics version 23.0, with significance set at *p *< 0.05.

## Results

### Participant demographics

The study cohort included 64 boys (46%) and 75 girls (54%) with a median age of 13.4 years, with 74% having sport-related injuries (Table 2). The median time from injury to study enrolment was 7.8 days (mean: 34.8 days, minimum: 2.9 h, and maximum: 320.9 days). Most participants were either straight A or A/B students prior to their concussion (29% were A and 37% were A/B). Fifty-six percent of participants (*N* = 76) had symptoms persisting longer than 1 month. The median time to full return to school time was 35 days, and the median return time to full sports competition was 38 days (19). Of note, our cohort intentionally included a heterogeneous sample of youth. The possible time from injury was *any time* within 1 year, assuming participants were still symptomatic (30).

**Table 2:** Participant demographics (*N* = 137).

Participant demographics	
Male	
*N* (%)	64 (46%)
Age	
Mean, *N* (SD)	12.9 (2.9)
Mechanism of injury, *N* (%)	
Sports/recreational play	103 (74%)
Non-sport-related injury/fall	22 (16%)
Assault	5 (4%)
Motor vehicle collision	4 (3%)
Other	3 (2%)
School information prior to injury	
Days missed in last 6 months, *N* (%)	
0	16 (12%)
1–2 days	37 (27%)
3–6 days	42 (31%)
7+ days	42 (31%)
Grade, *N* (%)	
1–6	52 (38%)
7–9	48 (35%)
10–13	38 (28%)
Average academic achievement prior to injury, *N* (%)	
Straight As	40 (29%)
As and Bs	51 (37%)
Straight Bs	23 (17%)
Bs and Cs	20 (15%)
Below C	2 (1%)
IEP prior to injury, *N* (%)	
Yes	30 (22%)

IEP, Individualized education plan.

### Participant recruitment

Some participants only had a First and Follow-Up Visit because their symptoms resolved in 28 days (*N* = 51). Participants who did not experience symptom resolution within the 6-month study period (*N* = 16) or achieved symptom resolution close to their scheduled Follow-Up Visit (*N* = 4) also only had a First and Follow-Up Visit. Participants who had symptoms for >28 days and experienced symptom resolution within the 6-month time period had an In-Person Symptom Resolution Visit (*N* = 44). Of the 100 participants who achieved symptom resolution within the study period (Figure 1), 44 completed the In-Person Symptom Resolution Visit and were provided the school information questionnaire. Twenty-six participants withdrew before symptom resolution could be determined.

### Aim 1: youth-reported perceived school problems

Of the 137 participants who consented to the study, 51% (*N* = 70) completed the school information questionnaire due to the age requirement (between 9.5 and 18 years old) and the time of study enrolment (after July 2015). Of these 70 participants, 63% (*N* = 44) had returned to school by the First Visit Post-injury Visit. Of the 44 participants who had returned to school, 50% (*N* = 22) reported experiencing school problems (new problems or problems that worsened since injury). Of those experiencing school problems (*N* = 22), the most common school problems were headaches (82%, *N* = 18/22), inability to pay attention in class (77%, *N* = 17/22), and being overly tired (73%, *N* = 16/22). Note that participants could select multiple answers, so data were not mutually exclusive.

Of the 44 participants who had returned to school by the First Visit Post-injury, 16% (*N* = 7/44) reported that their grades were affected. Grades were affected by 1 letter (e.g., A to B) for three participants and by 2 (e.g., A to C) or more letters (e.g., A to D) for two participants (Table 3). Of the 44 participants who had returned to school at the First Visit Post-injury, 55% (*N* = 24/44) reported experiencing trouble with classes (Figure 1). The most common subjects in which participants experienced difficulty were Mathematics (63%, *N* = 15), English (50%, *N* = 12), and Science (*N* = 10, 42%), compared to Art (4%, *N* = 1) or Music (25%, *N* = 6). At the First Visit Post-injury, 14 participants reported experiencing problems in both the morning and afternoon.

**Table 3:** School concerns and problems reported for youth with concussion at first visit, symptom resolution and follow-up visit.

School information	First visit	Symptom resolution	Follow-up visit
Return to school, *N* (% of total)			
Yes	44 (63%)	20 (87%)	65 (93%)
No	21 (30%)	1 (4%)	0
N/A	5 (3%)	2 (9%)	5 (7%)
Total respondents at each visit	70	23	70
School problems, *N* (% of total respondents at each visit)^a^			
Yes	22 (50%)	4 (17%)	12 (18%)
No	22 (50%)	16 (83%)	53 (82%)
Total	44	20	65
Grades affected, *N* (% of total respondents at each visit)^a^			
Yes	7 (16%)	3 (15%)	11 (17%)
No	8 (18%)	1 (5%)	39 (60%)
N/A	29 (66%)	16 (80%)	15 (23%)
Total respondents at each visit	44	20	65
Trouble with classes, *N* (% of total respondents at each visit)^a^			
Yes	24 (55%)	4 (20%)	12 (18%)
No	20 (45%)	16 (80%)	53 (82%)
Total respondents at each visit	44	20	65
School concerns, *N* (% of total respondents at each visit)			
Grades dropping	18 (26%)	4 (17%)	15 (22%)
Amount of work/falling behind	24 (35%)	4 (17%)	19 (28%)
Ability to learn, remember or concentrate	9 (13%)	7 (30%)	14 (21%)
Returning to sport	15 (22%)	7 (30%)	15 (22%)
Other	2 (3%)	1 (4%)	4 (6%)
Total respondents at each visit	68	23	67
Extent of concern, *N* (% of total respondents at each visit)			
Not concerned	11 (16%)	8 (35%)	39 (56%)
Mildly	26 (37%)	11 (48%)	18 (26%)
Moderately	22 (31%)	1 (4%)	7 (10%)
Very concerned	11 (16%)	3 (13%)	6 (9%)
Total respondents at each visit	70	23	70

^a^ Denotes a question that was only asked if participants responded yes to “Have you returned to school yet?”

Forty-four participants needed an In-Person Symptom Resolution Visit because they had persistent symptoms for >28 days (three visits in total) compared to the youth who recovered within 1 month post-injury (two visits). These participants also received a school information questionnaire, which 52% (*N* = 23/44) completed. Of the participants who completed the school information questionnaire (*N* = 23), 87% (*N* = 20/23) had returned to school. Of the 20 participants who had returned to school at the In-Person Symptom Resolution Visit, 20% (*N* = 4) reported experiencing school problems.

At the Follow-Up Visit, 70 participants completed the school information questionnaire, of which 93% (*N* = 65) had returned to school (Figure 1). Of the 65 participants who had returned to school at the Follow-Up Visit, 18% (*N* = 12) were experiencing school problems and 17% (*N* = 11) had their grades affected (Figure 1). Grades were most affected in English (36%, *N* = 4) and Science (36%, *N* = 4). Of the 65 participants who had returned to school at the Follow-Up Visit, 18% (*N* = 12) were experiencing trouble with classes (Table 3). The most common post-concussive difficulties for participants that led to difficulty with subjects were the inability to pay attention in class (75%), homework taking longer (75%), and difficulty understanding material (75%). At the Follow-Up Visit, of the 12 participants who reported school problems, 50% (*N* = 6) of participants reported experiencing class problems in both the morning and afternoon.

### Aim 2: youth-reported school concerns (type of concern and degree of concern)

At the First Visit and Follow-Up Visit, participants (Figure 1) reported that their greatest school concerns were the amount of work/falling behind, grades dropping, and returning to sport (Table 3). At the First Visit and Follow-Up Visit, participants reported their concern on how their injury would affect their learning and performance (Figure 1). The extent of this concern at the First Visit Post-injury was “mild” for 37% of participants (*N* = 26/70) and “very concerned” for 16% of participants (*N* = 11/70, Table 3). At the Follow-Up Visit, the extent of this concern was “none” for 56% of participants (*N* = 39/70), “mild” for 26% of participants (*N* = 18/70), and “very concerned” for 9% of participants (*N* = 6/70, Table 3).

### Parent-reported missed school

At each visit, parents were asked, *How many school days has your child missed since his/her injury?* At the First Visit, parents (*N* = 77) reported that their child missed 12.3 ± 16.6 days of school, whereas at the In-Person Symptom Resolution Visit, parents (*N* = 24) reported that their child missed 16.0 ± 17.7 days of school and at the Follow-Up Visit, parents (*N* = 74) reported that 16.2 ± 14.5 days of school were missed.

### Secondary aims: cognitive scale score and PCSS at stage entry and exit

**Figure 2:**
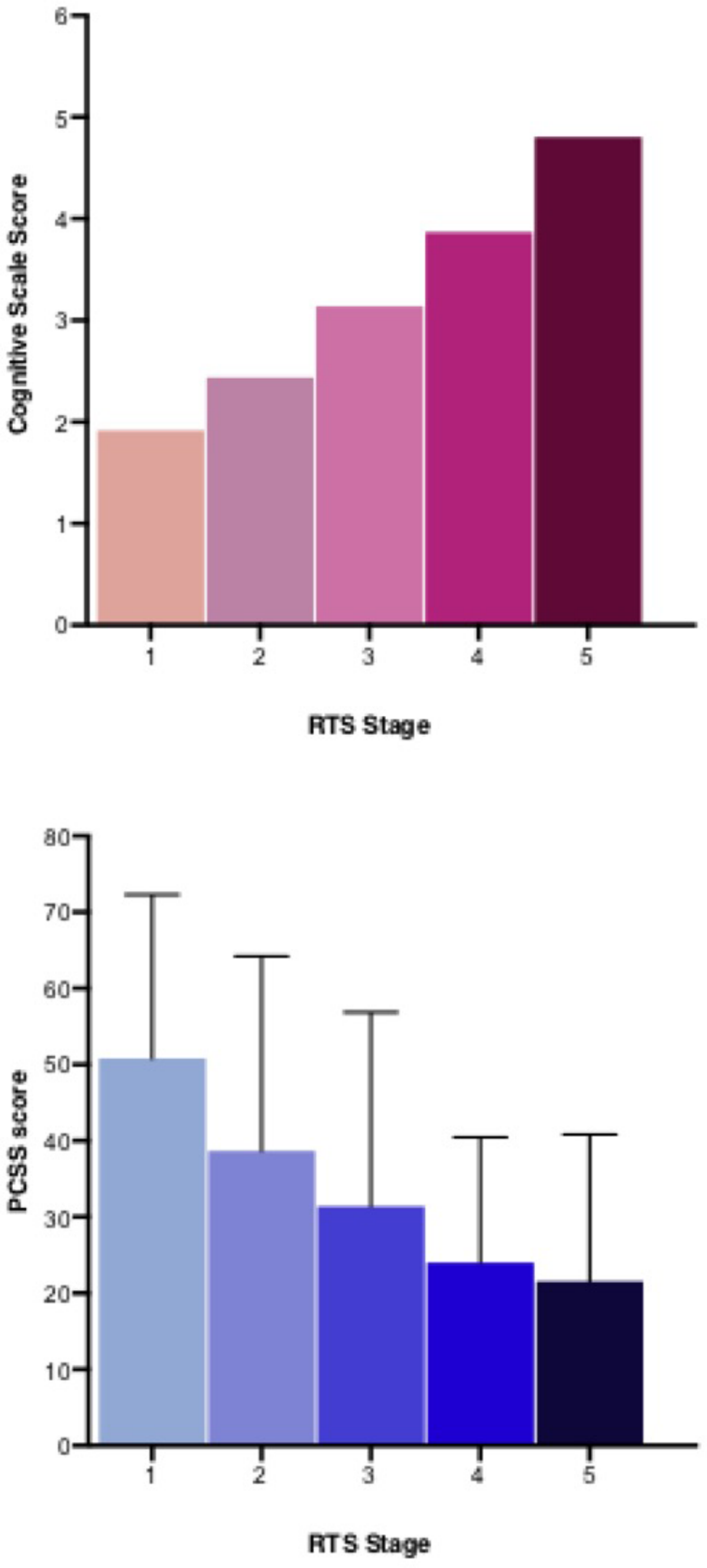
Post-concussion symptom scale (PCSS) and cognitive scale score at return to school stage entry.

There was a significant difference (*p *< 0.05) in the cognitive activity scale score for RTS stage entry and exit for Stages 2, 3, 4, and 5 (Table 4). The cognitive activity score increased from 2.0 ± 1.1 at RTS Stage 1 to 4.9 ± 0.2 at RTS Stage 5, corresponding to a full return to cognitive activity [Figure 2(1)].

**Table 4:** PCSS and cognitive scale score at RTS stage entry and exit (*N* = 107).

PCSS score															
Stage	1			2			3			4			5		
	Entry	Exit		Entry	Exit		Entry	Exit		Entry	Exit		Entry	Exit	
N	12	12		20	20		48	48		28	28		27	27	
Mean	50.8	49.8		38.7	27.9		31.4	21.9		24.0	18.5		21.6	17.4	
SD	21.5	27.1		25.5	23.5		25.5	16.3		16.5	14.4		19.3	16.6	
Min	17	9		5	4		5	2		1	2		2	1	
Max	83	100		94	79		118	66		53	72		72	57	
Z P			−0.55 0.58			−2.48 0.13			−3.75 0.00*			−2.53 0.01*			−1.95 0.05*
Cognitive scale score															
Stage	1			2			3			4			5		
	Entry	Exit		Entry	Exit		Entry	Exit		Entry	Exit		Entry	Exit	
N	38	38		48	48		94	94		95	95		107	107	
Mean	2.0 ± 1.1	2.2 ± 1.1		2.5 ± 0.8	2.7 ± 0.7		3.1 ± 0.9	3.5 ± 0.8		3.9 ± 0.9	4.3 ± 0.8		4.8 ± 0.5	4.9 ± 0.2	
Min	1	1		1	1		1	2		1	2		3	4	
Max	5	5		5	5		5	5		5	5		5	5	
Z P			−1.89 0.06			−2.55 0.01*			−4.14 0.00*			−4.17 0.00*			−3.66 0.00*

PCSS, post-concussion symptom scale; RTS, return to school.

Data were collected from 48 h and 2-week surveys. Only participants who reported a stage twice (entry and exit) were included in analysis. A Wilcoxon signed-rank test was performed for the difference between the PCSS score at entry and exit of each stage of the RTS protocols. A Wilcoxon signed-rank test was performed for the difference between the cognitive scale score at entry and exit of each stage of the RTS protocols.

* Statistically different from stage entry, p < 0.05.

The PCSS score decreased from 50.8 ± 21.5 at RTS Stage 1 to 17.4 ± 16.6 at RTS Stage 5 [Figure 2(2)]. There was a significant difference in the PCSS score at stage entry and exit for RTS Stage 3 (31.4 ± 25.5 to 21.9 ± 16.3 *p *= 0.01). Similarly, there was a significant difference in stage entry and exit at RTS Stage 4 (24.0 ± 16.5 to 18.5 ± 14.4) and Stage 5 (21.6 ± 19.3 to 17.4 ± 16.6) (Table 4).

### Secondary aims: ImPACT score at First Visit, Symptom Resolution, and Follow-Up Visit

The cognitive efficiency index, which is a subtest of the ImPACT and measures cognitive recovery following a concussion, increased from the First to Follow-Up Visit (0.25 ± 0.18 to 0.31 ± 0.15, *p *> 0.05, Table 5).

**Table 5:** ImPACT scores for youth with concussion (*N* = 113).

	First visit (*N* = 113)		Symptom resolution visit (*N* = 33)		Follow-up visit (*N* = 92)	
	Mean St Sc ± SD	Median	Mean St Sc ± SD	Median	Mean St Sc ± SD	Median
ImPACT						
Verbal memory composite score	79.6 ± 13.6	82.0	85.2 ± 8.4	87.0	85.9 ± 12.4	88.5
Visual memory composite score	68.5 ± 14.6	68.9	72.1 ± 12.7	75.0	72.3 ± 13.6	73.5
Visual motor speed composite score	30.0 ± 7.6	29.6	31.1 ± 7.9	33.4	33.1 ± 8.3	33.1
Reaction time composite score	0.72 ± 0.13	0.7	0.69 ± 0.12	0.67	0.67 ± 0.11	0.66
Impulse control composite score	8.7 ± 8.3	7.0	10.9 ± 13.2	8.0	9.4 ± 8.9	7.0
Cognitive efficiency index	0.25 ± 0.18	0.27	0.29 ± 0.21	0.33	0.31 ± 0.15	0.30

ImPACT, Immediate Post-Concussion Assessment and Cognitive Testing.

Immediate Post-Concussion and Cognitive Test: Pediatric ImPACT values (T-score) are very superior (70–80), superior (65–69), high average (58–64), average (43–57), low average (37–42), borderline (30–36), and impaired (28–29). Data were assessed for significance using a t-test.

### Secondary aims: parents vs. youth return to school and school problems

**Figure 3:**
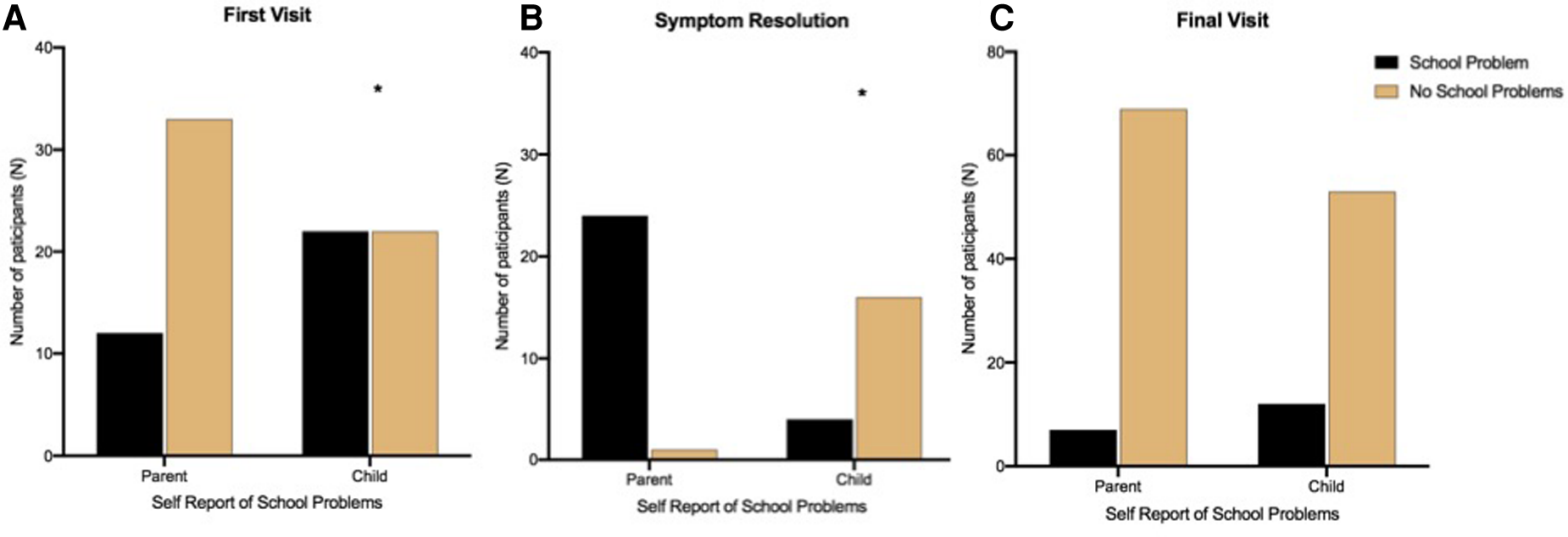
School problems reported by parents and youth with concussion.

At the First Visit, there was a significant difference (*p *= 0.02) in the school problems reported by parents (*N* = 45) and youth (*N* = 44) (Figure 3A). Here, 50% (*N* = 22) of youth reported school problems, whereas only 26% of parents (*N* = 12) reported that their child had school problems (Table 4). At the In-Person Symptom Resolution Visit, there was also a significant difference (*p *= 0.01) in the school problems reported by parents and youth (Figure 3B). Here, four youth (20%) reported school problems, whereas 96% of parents (*N* = 24) reported that their child had school problems (Table 6). No significant difference was observed between parents and youth at the Follow-Up Visit.

**Table 6:** Comparison of youth and parent report of school problems and concerns.

School information	First visit	Symptom resolution	Follow-up visit
School concerns			
Youth, *N* (%)			
Not concerned	11 (16%)	8 (35%)	39 (56%)
Mildly	26 (37%)	11 (48%)*	18 (26%)
Moderately	22 (31%)	1 (4%)	7 (10%)
Very concerned	11 (16%)	3 (13%)	6 (9%)
Total	70	23	70
Parents, *N* (%)			
Not concerned	8 (10%)	10 (40%)	36 (46%)
Mildly concerned	27 (34%)	6 (24%)	17 (22%)
Moderately concerned	22 (28%)	8 (32%)	12 (15%)
Very concerned	22 (28%)	1 (4%)	13 (17%)
Total	79	25	78
School problems, *N* (% of total)			
Youth, *N* (%)			
Yes	22 (50%)*	4 (20%)*	12 (18%)
No	22 (50%)	16 (80%)	53 (82%)
Total	44	20	65
Parents, *N* (%)			
Yes	12 (26%)	24 (96%)	7 (9%)
No	33 (73%)	1 (4%)	69 (91%)
Total	45	25	76

Chi-square tests were performed to determine school concerns and school problems for parents and youth.

* Statistically significant from parent report, p < 0.05.

Participants and their parents were also asked, *How concerned are you about this injury affecting your/your child's school learning and performance?* Participants and their parents reported similar concerns about school learning and performance at the First Visit Post-injury and Follow-Up Visit (Table 6). At the In-Person Symptom Resolution Visit, there was a significant difference (*p *= 0.04) in participants' and their parents' concern about the injury affecting their/their child's school learning and performance. Forty-eight percent of youth (*N* = 11/23) reported “mild concern” vs. 24% (*N* = 6/25) of parents (Table 6).

## Discussion

The aim of the present analyses was to describe school problems, school concerns, and cognitive activity patterns in youth during recovery from a concussion. Youth and their parents were assessed over time for perceived school problems as they recovered from their concussions. At the First Visit Post-injury (7.7 days), 50% of youth reported experiencing school problems, whereas only 18% of youth reported school problems at the Follow-Up Visit (133 days). Other literature reports high rates of perceived school problems, such as Ransom et al. (31), who examined academic problems among 349 concussed youth. The authors observed that 77%–88% of youth reported school problems as a result of concussion symptoms and diminished academic skills (31). This was much more prevalent in youth still experiencing concussion symptoms than those who had recovered, a pattern also seen in our cohort. Youth in our study perceived school problems in Mathematics, Science, and English/Writing classes. Symptoms of not being able to pay attention in class, being overly tired, homework taking longer, and difficulty understanding material all contributed to troubles in these classes. This is also similar to other findings in the literature, with Mathematics, reading/language, and Science being the most problematic (32).

Perceived school problems decreased across all three visits, with most problems being identified early on at the First Visit, so this suggests that issues at school generally improve over time. A small percentage of youth experience more significant problems such as a decrease in grades, which does not seem to change over time. Seventeen percent of participants, who prior to their injury were mostly straight A or A/B students, experienced a decrease in grades at the Follow-Up Visit. These findings are similar to other groups (33), including a scoping review (34) that reported a significant decline in academic performance following a concussion.

It is interesting that there is often some disagreement between parents’ and children's perceptions of academic problems (35). We observed a significant difference at two time points (*p *< 0.05), with the First Visit Post-injury difference being 50% of youth and only 26% of parents perceiving school problems. A reversal of this pattern was seen at the Symptom Resolution Visit with 20% of youth and 96% of parents identifying school concerns. Parent and child reporting about the same phenomena are often divergent (35), with no established or predictable directional reason for these differences.

In terms of concussion symptoms, children often report higher symptom levels than parents (36–38). Poor agreement between children and parents has also been observed for sleep problems (38) and neurocognitive symptoms (39, 40). To the best of our knowledge, only one study has investigated whether the child and parent report of symptom agreement varies over time. Liu and Hicks (40) observed that parents tended to report lower symptom severity in the first 2 weeks post-concussion and higher severity after 4 weeks (40). These findings are similar to our observations regarding school problems in which parents perceived fewer school problems at the First Visit Post-injury than the youth and more school problems at the Symptom Resolution Visit than youth. This reinforces that both should be sampled to get the whole family's perspective of what they are experiencing.

As anticipated, the amount and complexity of cognitive activity measured on a numeric scale increased with progression through RTS stages as symptoms decreased. This suggests that cognitive activity was modified according to protocols and symptom profiles. We also observed an increase in performance scores in all subtests of the ImPACT (i.e., verbal memory, visual memory, visual motor, impulse control, and cognitive efficiency index score) from the First to Follow-Up Visit. Similarly, Tjarks et al. (41) also observed an increase in ImPACT composite scores for youth aged 12–19 years over time, suggesting participants were progressively recovering from concussions. These results, an increase in the amount and complexity of cognitive activity as symptoms decrease, would be expected when a child was adhering to the RTS guidelines.

Despite several strengths, including longitudinal assessments (6 months following recruitment) and measures of cognitive activity and function, this investigation is not without limitations. First, data on race and socioeconomic status were not collected. Second, some participants were recruited during summer vacation or holiday break when the school questionnaire was not applicable. With this, we were unable to obtain school information from all participants. Third, our cohort intentionally included a heterogeneous sample of youth with varying time from injury *any time* within 1 year, as long as participants were still symptomatic (30). This meant the sample included youth with concussions experiencing both acute and prolonged symptoms due to the nature of the research question. As such, it is possible that the large variation in time to symptom resolution is due to the prolonged symptoms of some participants. Finally, although we were able to retain most participants, some were lost to follow-up or never achieved symptom resolution within the study period. Finally, there was no official record of preinjury school performance; this information was provided by parent and youth self-reports.

### Implications

Findings from this study may help guide youth recovering from a concussion and their parents as they return to school. We note the importance of returning to school cautiously and with accommodations to prevent a rise in symptoms that may be associated with an increase in cognitive activity. It is important that youth with a concussion and their parents monitor symptoms as they begin to increase their levels of cognitive activity to ensure that they are progressing through the stages at an appropriate rate. Also, given the significant difference between parent and youth reports of school problems, it is vital that parents and youth discuss these experiences with their healthcare providers to create a complete picture of the youth’s recovery. This finding is also important for future researchers, who should be encouraged to survey both parents and youth to capture all relevant information.

## Conclusions

Youths perceive a number of problems and concerns after concussions that are perceived to improve over time. Note that these are new problems and concerns that pre-date the youths’ concussion. A small percentage of youth experience more significant academic problems evidenced by a decrease in grades that do not improve over 6 months. There is a significant difference in the perception of school problems reported by youth and their parents at different stages of recovery. The amount and complexity of cognitive activity increased with decreasing symptoms and increasing RTS stage.

## Data Availability

The original contributions presented in the study are included in the article/Supplementary Material, further inquiries can be directed to the corresponding author.
